# Research progress on the application of transcranial magnetic stimulation in spinal cord injury rehabilitation: a narrative review

**DOI:** 10.3389/fneur.2023.1219590

**Published:** 2023-07-18

**Authors:** Yuhong Wang, Tingting Dong, Xiahuang Li, Huiyun Zhao, Lili Yang, Rui Xu, Yi Fu, Li Li, Xuesong Gai, Dongdong Qin

**Affiliations:** ^1^Department of Rehabilitation Medicine, The First People’s Hospital of Yunnan Province, Kunming, China; ^2^Department of Neurosurgery, Mengzi People’s Hospital, Mengzi, China; ^3^Department of Rehabilitation Medicine, Dongchuan District People’s Hospital, Kunming, China; ^4^Department of Pulmonary and Critical Care Medicine, Kunming Municipal Hospital of Traditional Chinese Medicine, Kunming, China; ^5^Department of Emergency Trauma Surgery, The First People’s Hospital of Yunnan Province, Kunming, China; ^6^Key Laboratory of Traditional Chinese Medicine for Prevention and Treatment of Neuropsychiatric Diseases, Yunnan University of Chinese Medicine, Kunming, China

**Keywords:** spinal cord injury, transcranial magnetic stimulation, rehabilitation, neuroplasticity, non-invasive brain stimulation

## Abstract

Traumatic or non-traumatic spinal cord injury (SCI) can lead to severe disability and complications. The incidence of SCI is high, and the rehabilitation cycle is long, which increases the economic burden on patients and the health care system. However, there is no practical method of SCI treatment. Recently, transcranial magnetic stimulation (TMS), a non-invasive brain stimulation technique, has been shown to induce changes in plasticity in specific areas of the brain by regulating the activity of neurons in the stimulation site and its functionally connected networks. TMS is a new potential method for the rehabilitation of SCI and its complications. In addition, TMS can detect the activity of neural circuits in the central nervous system and supplement the physiological evaluation of SCI severity. This review describes the pathophysiology of SCI as well as the basic principles and classification of TMS. We mainly focused on the latest research progress of TMS in the physiological evaluation of SCI as well as the treatment of motor dysfunction, neuropathic pain, spasticity, neurogenic bladder, respiratory dysfunction, and other complications. This review provides new ideas and future directions for SCI assessment and treatment.

## 1. Introduction

Spinal cord injury (SCI) has traumatic (e.g., car accidents and falls) and non-traumatic (e.g., infections and tumors) causes. Due to the limited repair ability of the central nervous system, SCI can lead to serious sensory, motor, and physical dysfunction below the injured segment. Moreover, SCI may cause neuropathic pain (NP), spasticity, neurogenic bladder, respiratory dysfunction, and other complications, which seriously affect the quality of life and life expectancy of patients (1).

With the growth of the global population, the prevalence and incidence of SCI remain high. In 2019, 900,000 incident cases, 20.6 million prevalent cases, and 6.2 million annual deaths related to SCI were registered (2). In China, the incidence of SCI ranges from 14.6 to 60.6 persons per million; furthermore, SCI incidence and health burden increase with time and age (3, 4). Expensive and complex medical support is needed after SCI. The lifetime financial burden of patients with SCI ranges from $1.5 million to $3 million (5). Meanwhile, the average annual health care cost incurred by patients with SCI in the United States is as high as $676,000 (6), which places a heavy economic burden on patients and the health care system. Therefore, SCI treatment and cost reduction remain a complex, global public health problem.

Protecting the nervous system and promoting neuron repair and regeneration are two primary directions for SCI therapy. In the early stage of SCI, the International Association of Neurorestoratology guidelines recommend surgical removal of fluid or tissue causing spinal cord compression, combined with the use of drugs to reduce inflammation and protect neurons (7). Subacute treatment mainly involves multi-disciplinary rehabilitation, with an extensive use of various treatments to improve neuroplasticity. Nevertheless, the efficacy of these treatments remains unclear.

In recent years, many scholars have found that neuromodulation methods, such as epidural spinal cord stimulation (8), transcutaneous spinal cord stimulation (tSCS) (9, 10), transcutaneous spinal direct current stimulation (tsDCS) (11), transcranial direct current stimulation (tDCS) (12), brain-computer interface-triggered functional electrical stimulation therapy (13), and transcranial magnetic stimulation (TMS) (14), can improve neuroplasticity, thereby potentially treating SCI. Epidural spinal cord stimulation and tsDCS can activate spinal neurons in specific segments and regulate spinal neural circuits (15). However, epidural spinal cord stimulation and tsDCS require surgical electrode implantation, and most patients do not accept invasive methods. In contrast, tSCS is a non-invasive stimulation technique. When the electrode is placed on the skin, it can stimulate spinal cord circuits and promote patient motor reflex response (9, 10). However, tSCS may be inaccurate in targeting specific areas of the spinal cord. Similarly, tDCS can stimulate neurons, but its spatial accuracy is limited, and thus connecting the stimulation with specific brain regions is challenging. In addition, brain-computer interface-triggered functional electrical stimulation therapy is complex, involves a high cost for research and development, and requires a high professional knowledge for operators; hence, its use in clinical practice is limited (16). TMS has the advantages of non-invasiveness, simple operation, and low cost compared with other neuromodulation methods. It can transmit a magnetic field through the scalp and skull using specific parameters to regulate neuronal activity in specific brain regions (17). Currently, TMS has become a primary means of non-invasive neuromodulation in patients with SCI.

**Figure 1:**
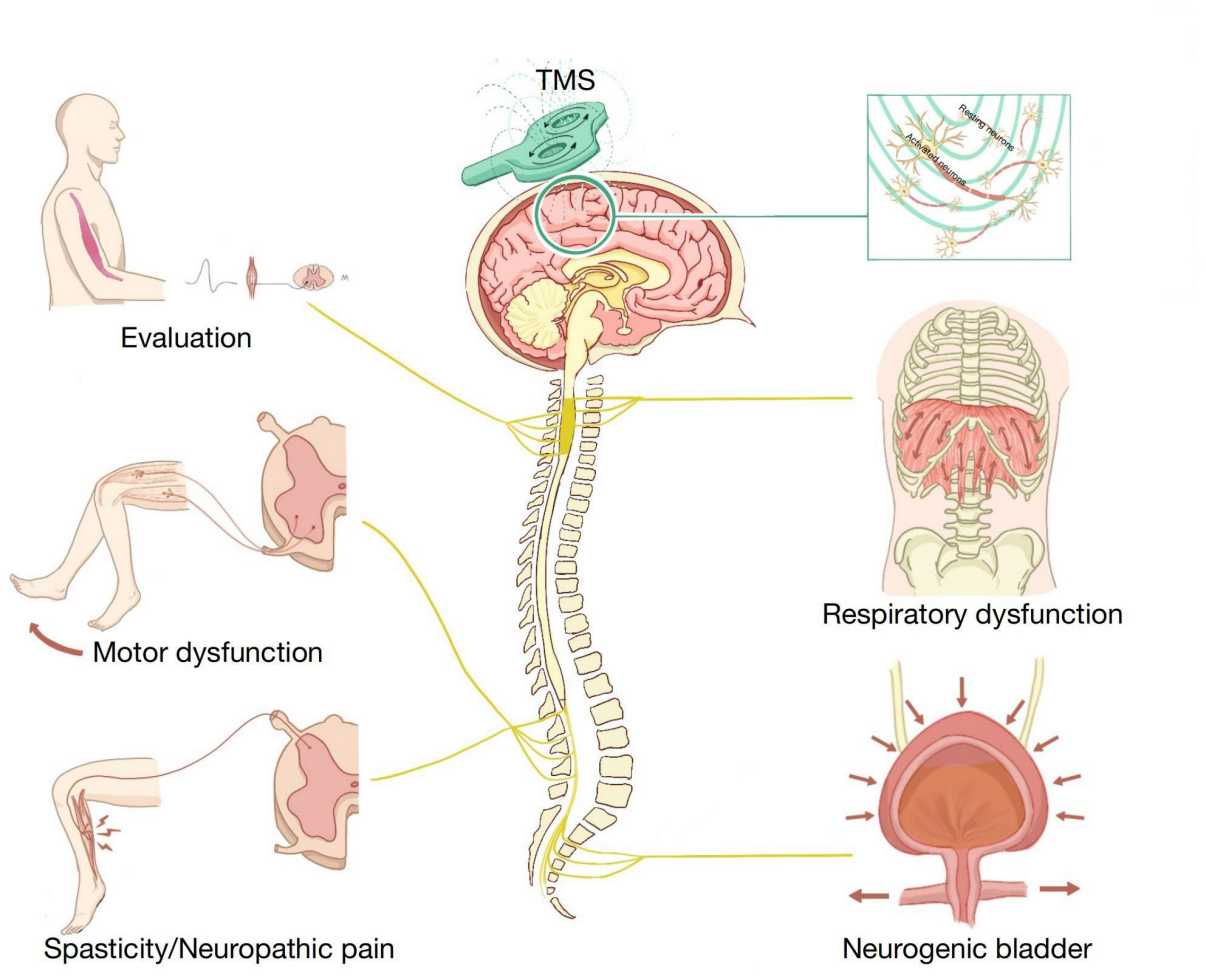
Application of transcranial magnetic stimulation (TMS) in spinal cord injury (SCI) rehabilitation.

Many studies have reported that TMS can improve synaptic plasticity and has a broad prospect in SCI treatment. TMS has been widely used in evaluating SCI and treating motor dysfunction, NP, spasticity, neurogenic bladder, respiratory dysfunction, and other SCI complications (Figure 1). In this article, we discuss the latest applications of TMS in SCI management. We used the PubMed database for a selective literature search of papers published between January 2000 and January 2023. Our key search terms were “spinal cord injury,” “SCI,” “transcranial magnetic stimulation,” “TMS,” “assessment,” “evaluation,” “motor dysfunction,” “neuropathic pain,” “spasticity,” “neurogenic bladder,” and “respiratory dysfunction.” Moreover, all the selected studies were written in English.

## 2. Pathophysiological mechanism of SCI

Understanding the pathophysiological changes that occur during SCI is the basis for making a treatment plan. SCI can be divided into two stages: primary and secondary SCI. Primary SCI involves spinal cord compression as well as shear, tear, or traction injury caused by spinal fracture, dislocation, and other factors, which can lead to local vascular damage, nerve parenchyma injury, ion imbalance, and glial membrane destruction (18). With further changes in physiological and biochemical reactions in the tissue, the spinal cord transitions into a stage of secondary injury.

Secondary SCI can be divided into acute, subacute, and chronic injury phases. In the acute phase, a large amount of calcium ions in the extracellular fluid flow into the cell, which leads to neuronal excitotoxicity, increases the concentration of reactive oxygen species and glutamate, and causes more severe damage to neurons and glial cells (18, 19). Moreover, the increase in cell membrane permeability causes inflammatory cells such as macrophages and microglia to infiltrate the injured site and release interleukin-6, interleukin-1β, and tumor necrosis factor-*α* to aggravate neuronal inflammatory response (20). Furthermore, spinal cord vascular injury leads to vascular ischemia, hypotension, and hypo-perfusion, resulting in cell death and tissue destruction (21). If the acute secondary injury is not effectively controlled, the body enters the subacute phase. Spinal cord changes during this period are characterized by neuronal apoptosis, axonal demyelination, Wallerian degeneration, axonal remodeling, and glial scarring (18, 21). Oligodendrocytes are essential in promoting axonal proliferation and myelination (22). However, during SCI, oligodendrocyte necrosis and apoptosis occur, leading to axonal demyelination, which affects axonal function and stability (23). When astrocytes proliferate to form glial scars, the injury starts healing and gradually transitions to the chronic phase. Eventually, massive cell death by apoptosis leads to cystic cavity formation, accompanied by axonal loss and glial scar maturation (24). In summary, pathological changes differ in different stages of injury, and therefore targeted treatment programs should be designed to promote nerve protection and regeneration.

## 3. Basic principles and classification of TMS

TMS is a non-invasive technique that can be used to stimulate specific brain tissues and affect the activity of local neurons by transmitting magnetic pulses from copper coils. The mechanism of TMS may be related to synaptogenesis or recombination as well as activation or inhibition of the activity of target cortical neurons, which leads to long-term synaptic potentiation or inhibition, thereby inducing changes in brain plasticity (25, 26). Tang et al. (27) found that TMS can change the density, loss rate, and formation rate of dendritic spines in adult and aged mice, in addition to changing the function of synaptic connections in the brain. Other studies have reported that TMS can improve neural plasticity by up-regulating the expression of brain-derived neurotrophic factor (BDNF), tropomyosin receptor kinase B, N-methyl-D-aspartate receptor, and synaptophysin (28). Moreover, TMS maintains the normal nerve conduction function by enhancing the myelination of demyelinated neurons (29). In addition, it can somewhat increase the survival and maturation of neonatal oligodendrocytes in the mouse cerebral cortex, resulting in neuroprotection (29). Furthermore, TMS appears to have some anti-inflammatory effects. It can increase the anti-inflammatory polarization of microglia to alleviate neuroinflammation and apoptosis; further, it promotes nerve tissue regeneration to a certain extent (30).

TMS was first used in the treatment of depression and other mental disorders. Many scholars have found that the application of TMS on the prefrontal cortex can effectively improve depression scores and clinical symptoms of patients with drug-refractory depression (31, 32). TMS is safe and well-tolerated; the incidence of epilepsy in patients undergoing TMS is less than 0.01% (10, 33). Therefore, in 2008, the United States Food and Drug Administration approved the use of TMS in drug-resistant depression treatment (34). Currently, TMS is used in the treatment of nervous system diseases such as SCI (35), Alzheimer’s disease (36), Parkinson’s disease (37), multiple sclerosis (38), stroke-related disability (39), and schizophrenia (40). TMS use has achieved gratifying results in basic research and clinical trials; hence, it is a potential treatment method for nervous system diseases.

TMS can be used as single-pulse stimulation, paired-pulse stimulation, repetitive TMS (rTMS), and paired associative stimulation (PAS). The single-pulse TMS program consists of single-pulse discharges that activate contralateral muscles when stimulating the primary motor cortex (M1). This activation can be recorded by electromyographic motor evoked potentials (MEP) (41). Paired-pulse TMS can be used to evaluate the excitability of intracortical or intercortical connections by paired stimulation (42).

rTMS is the most common stimulation protocol in therapeutic research, and different therapeutic effects can be produced when different parameters are used to stimulate the cerebral cortex. Generally, an rTMS frequency ≥5 Hz can increase the excitability of the motor cortex. In contrast, low-frequency rTMS (≤1 Hz) reduces cortical excitability, and thus it is used to regulate overactivity in specific brain regions (43). PAS includes the repeated pairings of single-pulse peripheral nerve electrical stimulation and single-pulse TMS of the corresponding motor cortex, which affects the cortical motor excitability of the corticospinal pathway to the target muscle (44). The excitability of the motor cortex is related to the interval between the two stimuli. The excitability of the corticospinal pathway increases when peripheral afferent stimulation is synchronized with TMS or reaches the motor cortex before TMS. Otherwise, corticospinal pathways will be inhibited (44–46). Nowadays, PAS has been shown to induce motor cortex plasticity and excitability (47).

Different coils have different penetration depths and stimulation ranges. The stimulation intensity of the circular coil is 0 in the center and maximum at the edge of the coil (48). The figure-eight coil can concentrate the stimulus at the center. Unlike the circular coil with an extensive stimulation, the figure-eight coil provides a more focused stimulation (48). However, both circular and figure-eight coils have shallow penetration depths. These coils can only be used to stimulate brain areas 2–2.5 cm from the scalp, and hence they are mainly used for cortical stimulation. To stimulate the deep brain regions, scholars have designed double cone, H-, and HCA coils, whose penetration depths can reach 3–6 cm (49). Therefore, different coils and stimulation protocols should be used for different treatment purposes in clinical applications.

## 4. Application of TMS in the rehabilitation of SCI

### 4.1. Evaluation

Clinically, the international classification of SCI is used to evaluate patient sensory and motor functions. Nevertheless, it only subjectively reflects the degree of injury and the lack of detection of changes in neurophysiological mechanisms. TMS is a method of neurophysiological examination, which can detect the residual corticospinal cord connection and nerve recovery after SCI; hence, it is a supplement to the international classification of SCI (50).

The MEP produced by applying TMS on M1 can detect the integrity of the descending conduction pathway from the cortex to the area below the injury level in patients with SCI; hence, it can be used to clinically recognize complete SCI. Although patients with complete cervical and thoracic SCI cannot elicit muscle activity during clinical examination, MEPs can be detected in trunk muscles, some lower limb muscles, and pelvic floor muscles during TMS (51, 52). Thus, there are residual corticospinal connections below the injury level, and rehabilitation should focus on the consolidation of these residual innervations. Evaluating the respiratory muscle function of patients with complete SCI above T6 remains a complex problem in clinical practice. Welch et al. (53) found that TMS combined with electromyography can be used to effectively characterize diaphragm activation in healthy individuals. More importantly, the MEP had good reproducibility in all participants, indicating that TMS-induced MEP may effectively detect cortical diaphragmatic pathway connectivity. In addition, TMS can be used to evaluate abdominal muscle function. Patients with complete SCI above T6 demonstrate abdominal muscle activity and maximal spontaneous (or attempted) contractions during TMS, suggesting that TMS can identify the preservation function of the corticospinal pathway by detecting abdominal muscle activity in patients with SCI (54).

TMS can be used to evaluate abnormalities of the corticospinal descending pathway. MEP latency represents TMS conduction time from the motor cortex to the peripheral nerve, including central motor and peripheral nerve conduction times. An abnormal MEP latency may reflect central and peripheral nerve conduction impairment. Nakamae et al. (55) used TMS to detect the MEPs of 831 patients with spinal cord lesions; the MEPs were recorded in the abductor digiti minimi and abductor hallucis muscles. They found that most patients had abnormal MEPs. Compared with the control group, 711 patients had prolonged MEPs, and 493 patients had prolonged central motor conduction times. A previous study tracked MEP changes in thenar muscles using motor cortex TMS from 19 to 1,109 days after injury in patients with incomplete cervical SCI (56). MEP latency was prolonged throughout the follow-up period, probably due to axonal injury, demyelination, and corticospinal tract degeneration (57). Apart from the changes in MEP latency, SCI may also decrease the MEP amplitude and increase the motor threshold (58), which somewhat reflect corticospinal conduction pathway abnormality. TMS can effectively assess the integrity of the corticospinal pathway in patients with SCI. The application of TMS in SCI evaluation is shown in Table 1. Future studies should enhance the accurate identification of SCI severity using TMS, which would strengthen its application in clinical practice and enable the development of targeted rehabilitation programs.

**Table 1:** Summary of clinical trials evaluating the efficacy of transcranial magnetic stimulation (TMS) for spinal cord injury (SCI).

Levels of SCI	ASIA scale	Sample size	TMS stimulated region	TMS protocol	Key outcomes	Reference
C4–T12	A–B	16	M1	Intensity: 50%–100% MSO	Muscles below the level of injury exhibit TMS-induced and/or autonomically induced activity	(51)
C6–T10	A–B	9	M1 representation of the PFM	Intensity: 60% MSO	The presence of MEPs in PFM induced by TMS indicates that possible preservation of descending pathways supplying the PFM	(52)
Healthy individuals	/	15	Left-hemisphere	Intensity: 60%–100% MSO	Diaphragmatic MEP induced by TMS and recorded *via* surface EMG can reflect cortico-diaphragmatic conduction	(53)
C5–T3	A	5	Abdominal region of M1	Intensity: 50%–100% MSO	Patients with SCI are able to activate the abdominal muscles in response to TMS and maximum voluntary (or attempted) contractions	(54)
Myelopathy	/	831	Vertex of the cranium	Intensity: 20% above the threshold for the MEPs	MEPs were prolonged in 711 patients (86%) and CMCTs were prolonged in 493 patients (59%)	(55)
C2–C7	C–D	21	Motor cortex	Intensity: 50% MSO	MEP latency was prolonged throughout the follow-up period in SCI patients	(56)
C2–C8	B–D	9	Motor cortex (hand)	Intensity: 110%–120% RMT	SCI patients have lower TMS evoked potential amplitudes and higher TMS motor thresholds	(58)

SCI, spinal cord injury; ASIA, American Spinal Injury Association; TMS, transcranial magnetic stimulation; M1, primary motor cortex; MSO, maximal stimulator output; MEP, motor-evoked potential; PFM, pelvic floor muscle; EMG, electromyography; RMT, resting motor threshold; CMCT, central motor conduction time.

### 4.2. Treatment

#### 4.2.1. Motor dysfunction

Motor dysfunction causes independence loss and quality of life decline in patients with SCI. Thus, motor function recovery is the primary goal of rehabilitation. A previous study explored the efficacy of rTMS in the treatment of four patients with incomplete cervical SCI. After continuous motor cortical stimulation for 5 days, TMS significantly improved the upper limb motor function, motor score, and pinprick sensory score (59). This effect may be related to increased motor cortical excitability and decreased corticospinal tract inhibition by rTMS. In addition, rTMS improves lower extremity motor function. Benito et al. (60) found that rTMS with a frequency of 20 Hz, 1800 pulses, and 90% resting motor threshold intensity for 15 days could improve the lower limb function score and gait function of patients with SCI, and the effect could be maintained for 2 weeks after treatment. Similarly, another study reported that routine rehabilitation training combined with rTMS can improve lower limb muscle strength in patients with SCI (35). When the motor cortex of the lower extremities was exposed to rTMS at 20 Hz and 1800 pulses for 4 weeks, the maximum muscle strength of knee flexion and extension in the combined treatment group was significantly higher than that in the routine rehabilitation group. An increasing number of researchers have found the advantage of combination therapy in neural circuit regeneration and reconnection. Increasing the plasticity of the corticospinal junction may be the key to improving motor function after SCI. Wang et al. (61) compared the efficacy of rTMS combined with treadmill training with that of treadmill training only or rTMS intervention only. The motor and coordination functions of rats in the combined treatment group was significantly increased, and the curative effect was better than that of the treadmill or rTMS group. BDNF, synaptophysin, and postsynaptic density protein 95 levels in the cortex and spinal cord were significantly increased in the combined treatment group, suggesting that the combined therapy promoted the plasticity of the motor cortex and spinal cord. The effect of rTMS or treadmill training on spinal cord plasticity is limited; rTMS can only improve the plasticity of the cortex. Although treadmill training can increase the expression of BDNF, it cannot effectively induce the expression of synaptophysin and postsynaptic density protein 95.

In addition to the traditional rTMS protocols, some new stimulation protocols have proven to improve motor dysfunction after SCI. Intermittent theta burst stimulation (iTBS) is a particular scheme of TMS that can induce a long-term potentiation of the motor cortex; iTBS for 190 s can excite the cortex for 60 min (62). Marufa et al. (63) explored the effect of the iTBS protocol on rats with incomplete SCI. After 2 weeks of stimulation, the motor function score and MEP amplitude were significantly increased, and the expression of growth-associated protein 43 (GAP43) in the spinal cord was significantly up-regulated. GAP43 expression is closely related to synaptic formation. GAP43 expression up-regulation promotes innervation and neurogenesis in damaged areas (64), suggesting that TMS promotes axonal regeneration by up-regulating GAP43 expression, thereby improving the motor function of rats. Traditional rTMS at 20 Hz combined with tsDCS (rTMS-20 Hz/tsDCS) can be used to treat brain and spinal cord lesions simultaneously and is advantageous in neuroplasticity improvement. A study compared the efficacy of rTMS-20 Hz/tsDCS and rTMS-iTBS/tsDCS in patients with chronic SCI and found that both stimulation protocols significantly improved MEP latency, MEP amplitude, and lower limb muscle strength score (65). Some recent studies have reported that nerve root magnetic stimulation can improve motor function, enhance nerve conduction, and promote the recovery of synaptic ultrastructure in the sensorimotor cortex of SCI rats (66). Based on the gratifying results of animal experiments, other studies explored the effect of combined magnetic stimulation of the nerve root and cortex on the lower limb motor function of patients with SCI (67). Table 2 shows the results of clinical trials on the efficacy of rTMS in patients with post-SCI motor dysfunction.

**Table 2:** Summary of clinical trials on the efficacy of TMS for treating SCI-induced complications.

Levels of SCI	ASIA scale	Sample size	TMS stimulated region	TMS protocol			Treatment cycle	Key outcomes	Reference
				Frequency	Intensity	Number of pulses			
*Motor dysfunction*									
C5	D	4	Left motor cortex	Double pulses (0.1 Hz/10 Hz)	90% threshold	360 doublet pulses	10 days	RTMS can alter cortical inhibition in incomplete SCI and improve the clinical and functional outcomes	(59)
C4–T12	D	7	Leg motor area of brain	20 Hz	90%RMT	1,800	3 weeks	High-frequency rTMS can improve spasticity, motor function, and gait in motor incomplete SCI	(60)
C2–L2	A–D	11	Bilateral leg motor cortex	20 Hz	100% RMT	1,800	4 weeks	Great improvement in lower limb MVC and LEMS in rTMS group	(35)
C2–T11	B–D	9	Vertex of brain	① rTMS-20 Hz ② rTMS-iTBS	90% RMT	① 1,600 ② 600	1 day	Paired stimulation in both groups significantly improved MEP latency, MEP amplitude, and LEMS in chronic SCI subjects	(65)
C–D	C–D	110	① M1 ② L3/L4	10 Hz	100% RMT	1,000	4 weeks	Clinical study protocol, no results	(67)
*Neuropathic pain*									
C1–T4	A–D	16	Hand/leg M1 area		90% RMT	2,000	1 day	RTMS applied over the hand or leg motor cortex decreased NP	(68)
C3–L1	A–D	14	Left M1	10 Hz	80% RMT	1,200	6 weeks	High-frequency rTMS effectively enhances the analgesic effects on neuropathic pain after SCI	(69)
C4–L5	A–D	24	Hand area of M1	10 Hz	90% RMT	1,500	3 weeks	rTMS relieves acute neuropathic pain in patients with SCI	(70)
C5–T10	A–D	6	PMC DLPFC	10 Hz	120% RMT	1,250	2 weeks	RTMS may be effective in alleviating NP in SCI patients	(71)
NP	/	18	M1	5 Hz	90% RMT	500	10 days	Pain was significantly improved after deep rTMS with H-coil	(72)
NP	/	50	M1 DLPFC	10 Hz	115% RMT	1,250	4 weeks	Clinical study Protocol, no results	(73)
*Spasticity*									
C4–T12	C–D	14	Left primary motor cortex	20 Hz	90% RMT	1,600	5 days	rTMS improved spasticity in patients with incomplete SCI, and MAS and MPSFS were significantly reduced	(74)
C5–T8	C–D	10	M1 (leg area)	iTBS	0% AMT	600	10 days	Resting and active MEP amplitudes were significantly increased and spasticity was reduced in SCI patients	(75)
C5–T10	C–D	8	Left M1	20 Hz	90% RMT	1,600	5 days	rTMS can decrease lower limb spasticity and restore impaired excitability in the disynaptic reciprocal inhibitory pathway	(76)

SCI, spinal cord injury; ASIA, American Spinal Injury Association; TMS, transcranial magnetic stimulation; rTMS, repetitive transcranial magnetic stimulation; RMT, resting motor threshold; MAS, modified Ashworth scale; MVC, maximal muscle strength; LEMS, lower extremities motor score; MEP, motor-evoked potential; M1, primary motor cortex; NP, neuropathic pain; PMC, premotor cortex; DLPFC, dorsolateral prefrontal cortex; MAS, modified Ashworth scale; MPSFS, modified Penn Spasm frequency scale; iTBS, intermittent theta burst stimulation; AMT, active motor threshold.

#### 4.2.2. NP

NP is a common complication after SCI. A meta-analysis showed that approximately 53% of patients with SCI develop NP (77). Although drugs such as gabapentin and pregabalin can somewhat relieve NP, only 30%–50% of patients experience pain alleviation (78). The latest evidence-based guidelines suggest that rTMS can relieve pain to some extent, and its analgesic effect is grade A (clearly effective) (79). rTMS may be a new alternative treatment for NP. Previous studies have found that rTMS on M1 can reduce NP in patients with SCI, and a significant analgesic effect is obtained within 48 h after the first treatment (68). Furthermore, Sun et al. (69) found that high-frequency rTMS on M1 can enhance the analgesic effect of conventional rehabilitation and drug therapy on NP. As shown by functional near-infrared spectroscopy, it was also observed that rTMS suppressed M1 and premotor cortex activation, which may account for pain relief. In addition, rTMS can relieve acute NP. A study showed that rTMS at 10 Hz in the hand region of the motor cortex could reduce acute NP in the early stage of SCI, improve MEP-related parameters, and regulate the secretion of BDNF and nerve growth factor (70). Moreover, the analgesic effect of rTMS on the abovementioned parameters can last for 2 to 3 weeks. In addition to M1, the prefrontal cortex may also be an effective target for NP treatment after SCI. Nardone et al. (71) stimulated the prefrontal cortex of patients with SCI using 10 Hz rTMS. NP was significantly alleviated after ten courses of treatment; the mechanism of pain relief may be related to the activation of the anterior cingulate gyrus and pain control circuits as well as the release of endogenous opioids during rTMS (12, 80). However, current research findings cannot explain the best target of rTMS for NP treatment, and a comparative study on the effects of M1 and prefrontal cortex stimulation is in progress (73).

In addition to the stimulation targets, other studies compared the efficacy of deep rTMS using H- and figure-eight coils on NP. The results showed that M1 stimulation using the H-coil significantly relieved lower extremity NP, and the visual analog scale score decreased significantly after 1 h of stimulation. In contrast, stimulation using the figure-eight coil did not improve pain (72). Moreover, the frequency, intensity, and number of treatments affected the efficacy of rTMS. Table 2 presents the findings of clinical trials on the effects of rTMS on NP after SCI. However, the current stimulation parameters vary differently with rTMS, and the times for different stimuli to produce curative effects are uneven. Further research is required to evaluate the effective stimulation parameters as well as the short- and long-term curative effects of rTMS on NP.

#### 4.2.3. Spasticity

The prevalence of spasticity after SCI is as high as 65%, and nearly 35% of patients need intervention (81). Medications such as baclofen are commonly used to manage spasticity. However, the long-term use of baclofen can produce side effects such as sedation, lethargy, ataxia, and decreased muscle activity, with limited efficacy in spasticity improvement (82). Previous studies have shown that high-frequency rTMS can reduce spasticity in patients with multiple sclerosis or stroke (83, 84); hence, rTMS may be a new method of relieving spasticity. Kumru et al. (74) stimulated the M1 of patients with SCI using 20 Hz rTMS. After 5 days of intervention, rTMS significantly improved the lower extremity spasticity of 15 patients. Therefore, the use of rTMS to improve spasticity after SCI is safe and feasible. Similarly, in the iTBS protocol, rTMS reduces the modified Ashworth scale score (an SCI assessment tool for spasticity) and the H/M amplitude ratio of the soleus H reflex in patients with SCI, thereby effectively relieving spasticity (71, 75). Notably, rTMS can maintain the effect of this treatment protocol for 1 week after the end of treatment. RTMS can reduce spasticity and create more conditions for the rehabilitation of patients with SCI, which is beneficial for muscle function consolidation and functional recovery promotion.

In addition, combination therapy is effective in improving spasticity. A recent study found that rTMS combined with treadmill training reduced hyperreflexia, improved motor function, and increased the expressions of K^+^-Cl^−^ cotransporter 2 (KCC2) and glutamic acid decarboxylase 67 (GAD67) in SCI rats (85). The up-regulation of KCC2 expression in the membrane of motor neurons reduces post-SCI spasticity (86). GAD67 expression is strongly correlated with GABA levels in spinal cord inhibitory synapses, and GABA expression inhibits neuronal overexcitation (87). Increasing KCC2 and GAD67 expressions can increase the inhibitory input to motor neurons and rebalance the excitability of motor neurons, thereby improving motor function. In addition to the known molecular mechanisms, some scholars have explored the possible physiological mechanisms by which rTMS relieves spasticity. Nardone et al. (76) evaluated the mutual synaptic inhibition of type Ia motor neurons of the soleus muscle in patients with SCI and concluded that rTMS may reduce segmental spinal cord excitation by enhancing the descending projection between the motor cortex and inhibitory spinal cord neuronal circuit, thereby reducing leg spasticity. This effect reverses the loss of mutual inhibition after SCI and improves the cerebral control of the spinal cord. Table 2 shows the findings of clinical trials investigating the effect of rTMS on spasticity after SCI.

#### 4.2.4. Neurogenic bladder

The sacral voiding center (S2–S4), pontine micturition center, and cerebral cortex are responsible for promoting and inhibiting micturition and maintaining the urinary function. Generally, sympathetic nerves (T10–L2) provide inhibitory input to the bladder, leading to bladder filling. Moreover, bladder emptying is caused by excitatory inputs supplied by parasympathetic nerves (S2–S4) (88). When SCI occurs, communication between the brain and the spinal nerves that control the bladder may be interrupted, resulting in bladder and urethral sphincter dysfunction, leading to the development of a neurogenic bladder (88). Approximately 70% of patients with SCI have neurogenic bladder (89). Neurogenic bladder causes urinary incontinence or retention. It may also be complicated by urinary tract infection as well as kidney and bladder stones, which seriously affect patient quality of life (90). Therefore, improving bladder function significantly improves the quality of life of patients with SCI. rTMS has been shown to regulate the activities of pelvic floor and bladder muscles, and hence rTMS may be an adjuvant therapy for bladder function improvement (91). Jang et al. (92) also found that the left anterior cingulate gyrus is directly related to micturition initiation and coordination. Activating the left anterior cingulate gyrus using rTMS may enhance the functional recovery of patients with bladder sphincter and reflex disorders. Another study explored the efficacy of 1 Hz rTMS of the bilateral dorsolateral prefrontal cortex on bladder pain syndrome treatment (93). At the end of the rTMS regimen, suprapubic pain disappeared completely, and the micturition frequency decreased by 60%–80%, indicating that rTMS can potentially improve bladder function. Moreover, a previous study compared the effects of high- and low-frequency rTMS of the supplementary motor area of the brain on pelvic floor muscle activity and reported that high-frequency rTMS can inhibit pelvic floor muscle activity, and thus may be used to relieve pelvic floor pain (94). In contrast, low-frequency rTMS can increase pelvic floor muscle activity, which may be an effective method of improving urinary incontinence. The two schemes are advantageous in neurogenic bladder management; hence, the appropriate scheme should be selected based on the patient’s condition. Similarly, Brusa et al. (95) reported that low-frequency rTMS of the pelvic floor motor cortex can increase bladder capacity and sensation during the first filling period and reduce bladder overactivity. The mechanism of bladder function improvement may be related to the reverse regulation effect of rTMS on the descending pathway of the corticospinal tract projecting to the detrusor muscle. Nevertheless, a previous study reported that 5 Hz, high-frequency rTMS of the primary motor cortex with a threshold intensity of 100% significantly improved bladder dysfunction during micturition (96). This suggests that high-frequency rTMS may improve detrusor contraction and/or urethral sphincter relaxation by enhancing the excitability of the corticospinal tract. Although previous studies have reported that rTMS can potentially affect the improvement of bladder function in patients with multiple sclerosis-related bladder disease, Parkinson’s disease-related bladder disease, bladder pain syndrome, and other related diseases, the effect of rTMS on post-SCI neurogenic bladder treatment remains unclear (97). Further studies are required to clarify this effect.

#### 4.2.5. Respiratory dysfunction

Patients with high cervical SCI usually develop apparent respiratory muscle paralysis, accompanied by cough reflex and mucociliary clearance disorders, which can cause complications such as pneumonia and respiratory failure in severe cases (98, 99). Therefore, managing post-SCI respiratory dysfunction remains a complex clinical problem. Previous studies have found that magnetic stimulation activates the diaphragm and regulates diaphragmatic excitability by activating the remaining phrenic nerve circuit after cervical SCI (100). Michel-Flutot et al. (101) confirmed that a single high-frequency (10 Hz) rTMS could persistently increase the excitability of the phrenic neural network in anesthetized rats. Moreover, inhibitory rTMS on the supplementary motor area was found to suppress the excitability of the corticospinal pathway to the diaphragm in healthy individuals. Furthermore, high-frequency rTMS can reduce hyperventilation and change the respiratory pattern during inspiratory threshold loading (102, 103). Therefore, rTMS may be a new way to improve respiratory dysfunction after SCI. Michel-Flutot et al. (104) investigated the effects of rTMS on respiratory function at 7 days, 1 month, and 2 months of treatment in rats with C2 spinal cord hemisection. The results showed that regardless of the intervention time, rTMS had no significant effect on the diaphragm activity of the injured side. However, rTMS increased the diaphragm activity of the normal side at 1 month and strengthened the existing cross-phrenic nerve pathway at 2 months, which increased the diaphragm activity of the injured side during asphyxia. This effect may be due to the increased expression of GAP43-positive fibers that enhance the respiratory descending fibers in the ventrolateral funiculus, which in turn induces the plasticity of phrenic nerve cells. Additionally, the results suggest that rTMS alone may not be sufficient to stimulate significant diaphragmatic function recovery. It should be used in combination with other treatments to enhance its efficacy. A randomized controlled trial compared the effects of rTMS combined with respiratory training and respiratory training alone on pulmonary function in patients with ischemic stroke. Combined therapy significantly improved the pulmonary function of patients with ischemic stroke, and the effect was significantly better than that of patients in the respiratory training group (105). Therefore, rTMS seems to be an auxiliary method of respiratory function improvement. However, these results should be carefully considered due to the limited number of related studies. A large number of studies are needed to confirm the efficacy of rTMS in respiratory dysfunction management.

## 5. Conclusion and prospects

SCI causes severe sensorimotor dysfunction by damaging the corticospinal system, often resulting in lifelong disability. In addition, SCI causes an imbalance between cerebral cortical excitability and inhibition (106). Regulating the imbalance between excitability and inhibition of cortical and corticospinal tract networks may be the focus of SCI treatment. A previous study reported that dual tDCS inhibition the unaffected lateral hemispheres of patients with stroke through cathodic stimulation, thereby reducing its inhibition on the affected lateral hemispheres (107). Subsequently, the affected hemispheres were activated by anodic stimulation, which increased their MEPs, thereby improving the balance of cerebral excitability and inhibition at the central level. TMS can also affect cortical excitability and inhibition at the central level. On the one hand, TMS can improve the motor function of patients with SCI by increasing corticospinal tract excitability. On the other hand, TMS can inhibit abnormal cortical excitability to relieve spasticity and NP. Therefore, the selection of excitatory and inhibitory parameters should be determined according to the specific conditions of the patients and the specific time after the injury.

Currently, rehabilitation has focused on reducing complications, improving the dysfunction, and ameliorating the quality of life of patients with SCI. As a non-invasive tool that might influence cortical excitability, TMS has demonstrated some curative effects on SCI and its complications, such as motor dysfunction, NP, spasticity, neurogenic bladder, and respiratory dysfunction. TMS may be a new method for SCI evaluation and treatment. Different TMS treatment schemes can alleviate various complications at the same time (35, 108). Furthermore, TMS can be used as an adjuvant treatment to enhance the efficacy of the initial treatment (69, 109). However, TMS is mostly applied in SCI management *via* basic research and experimental clinical studies; more research is required to promote its clinical application. In addition, the TMS regimens used in most studies are different; hence, the appropriate stimulation targets and parameters remain unclear. Notably, the effects of TMS on neuroplasticity in the central nervous system require further clarification, as the exact mechanisms remain unelucidated. Therefore, more large-scale, multicenter randomized controlled trials should be conducted to determine the efficacy and safety of TMS. Moreover, the mechanism of TMS on SCI rehabilitation should be continuously explored. Some non-invasive brain stimulation techniques such as tDCS have been proven to relieve spasticity and NP caused by stroke, multiple sclerosis, SCI, and other neurological diseases; furthermore, these techniques somewhat improve patient motor function (110, 111). Scholars should carefully consider these results and design more clinical trials to compare the efficacies of TMS and tDCS in SCI management.

## Author contributions

All authors listed have made a substantial, direct, and intellectual contribution to the work and approved it for publication.

## Funding

This work was supported by the National Natural Science Foundation of China (82160442, 31960178, and 82160923); Applied Basic Research Programs of Science and Technology Commission Foundation of Yunnan Province (2019FA007), and Kunming Medical University Joint Special Project (2019FE001(-300)).

## Conflict of interest

The authors declare that the research was conducted in the absence of any commercial or financial relationships that could be construed as a potential conflict of interest.

## Publisher’s note

All claims expressed in this article are solely those of the authors and do not necessarily represent those of their affiliated organizations, or those of the publisher, the editors and the reviewers. Any product that may be evaluated in this article, or claim that may be made by its manufacturer, is not guaranteed or endorsed by the publisher.
